# Extraction of Teeth

**Published:** 1853-01

**Authors:** J. Taylor


### *Extraction of Teeth.*—By Dr. J. Taylor.

We propose giving two or three short articles on this subject, giving a description of some of the forceps and other instruments we use in this operation, their mode of application, &c.

This operation we are glad to say, has lost much of its terrors in the last few years. This is mainly attributable to the improvements which have been made in the instruments used, and these improvements have special reference to the forceps. When we commenced extracting teeth (in 1827,) the key was *the great* instrument. This was used by Dentists and Physicians, to almost the exclusion of forceps and every thing else. We confess we have a kind of reverence for the old lion which has been kicked out of our cases and lays rusty and stripped of his glory in some old drawer, along with a few fragments of hippopotamus tusks. Occasionally we bring him forth, and rub him off, and give him a taste of battle and blood again. We almost conceive he feels gratified at the little notice we thus pay him. We have always thought he was very tame and tractable in our hands, for he never treated us shabbily but once; and to make the case more aggravating, he took advantage of us when very weak and feeble from an attack of pleurisy; he then jumped clear out of our hands, also out of the patient's mouth, and with his *claw* wounded us on the forehead. We know not, if, we could ever have forgiven him this rudeness, but the patient happened to be a doctor, and we suppose he had abused the tribe, and he wished to take revenge on him, and our wound was a mere accident, being somewhat in his way. Out of respect for his memory we shall first introduce him to our readers. He has an ivory handle with a silver band around either end; his body is curved a little like the camel, with the bump flattened and elongated, this throws the handle a little lower than it would otherwise be and brings it on a line with the fulcrum. Originally he had a snout something like the elephant, extending some quarter of an inch beyond the fulcrum; this was arranged for the adaptation of a hook or *claw*; but we thought it a useless appendage and cut it off. The fulcrum did not suit us either, for it gave too much latitude to his rotary movements, so we filed it down, flattening the sides so that they were adapted to the rounded labial and palital face of the teeth. The most depending portion we rounded off, and thus reduced somewhat the length. The claws (for he had five or six) we sharpened, and the largest one we trimmed to fit between the bifurcated labial roots of the superior molars. The next in size we similarly adapted to the inferior molars. His smaller *claws* we grooved and adapted to fit the roots of the bicuspids. For the front teeth he appeared never to have a liking, and we felt no disposition to enforce a taste so entirely repugnant to his nature. What is a little remarkable, he always showed *his wisdom* in wishing to avoid the *wisdom* teeth. Whether this was owing to the deprivation of his snout we know not, yet being the only ungovernable part about him, we consulted our own taste in its removal.

Having thus tamed and trimmed this remorseless tyrant, we give him the use of but one *claw* at a time, and to make his application a little more easy, we take a fine napkin or silk handkerchief, and after doubling pass the corner or end about twice around the fulcrum, and then neatly roll it back on the bar or handle ; thus harnessed he is fit for duty.

In the application of the turnkey some diversity of opinion exists; some generally applying the fulcrum on the outside or labial portion of the teeth ; others generally reversing this order. The latter we prefer as a general rule ; and think it most in accordance with the position of the teeth, and the direct application of the force for their removal. The upper margin of the alveolus, which surrounds the teeth in the inferior maxilla, is generally on a higher level on the labial than palital face of the teeth. This is true also in relation to the teeth of the superior maxilla. In the use of this instrument, it is well to be remembered, that we cannot much change that force being applied for the removal of the teeth. There is less resistence when the force is so applied as to draw from the higher edge of the alveolus. The under teeth incline, generally, a little inward ; and when the fulcrum is placed on their palital face, the power applied with the hook more directly draws the tooth on a line with the direction of the alveolus. The lower down upon the neck of the tooth the hook is placed the better. In the use of this instrument, the great difficulty generally is, that the fulcrum gets too low for the point of the hook. When this takes place, the application of force does not raise the tooth direct from the socket; but pulls it transversly across, forcing open the alveolus, and tearing the teeth out by fracturing the alveolus; or as is often the case, breaking the teeth off. We remarked that we had flattened the sides of our fulcrum. This was done, so, that as the first turn of the instrument was made, the back of the fulcrum should throw in, on to the upper margin of the alveolus, against the neck of the tooth.

The manner in which we hold the head of our patient, the instrument, &c., is as follows. In the extraction of the under teeth, we stand to the right, and a little before the patient. Sometimes, but not often, we may take our position on the left. The head is kept about strait with the body, and not thrown back. The palm of our hand always is on top of the handle; and not thrown under; which turns the arm, and does not give unrestrained motion of the muscles. This position of the hand, it will be seen, renders it necessary to use either hand, for the extraction of the teeth. On the left side, the left hand takes hold of the handle of the instrument; and for the right side, the right hand. A little practice will make this familiar, and perfectly easy. We prefer holding the handle of the key in our left hand; although not left handed. This is because we regard the position and use of the other, as equally important. We are about applying the key to a left inferior molar; the left hand grasps the handle of the instrument; we are standing a little in front, and to the right of our patient; the palm of our right hand (or rather fingers,) is placed under the chin and inferior maxillary bone; the thumb is passed inside of the lip, and rests upon the top of the hook; and we embrace firmly between the fingers and the thumb, the jaw and the hook of the instrument. We are thus enabled to press the hook down to the edge of the alveolus, and with the fore-finger of the left hand, guide the bar of the instrument in the adjustment of the fulcrum. The key, when thus adjusted and held, can scarce fail to act properly; and gives us perfect mastery over our patient; so that the head cannot be jerked from us; or the instrument thrown off the tooth. This arrangement, also gives stability to the moveable lower jaw. The adjustment is reversed for the right side; so that the thumb or forefinger always rests on the hook of the instrument. We first feel that the hook is put to its right place; and we hold this to its place until the tooth is removed. This enables us also to bring with ease, the fulcrum to its place; and feel assured that the entire adaptation of the instrument is right. As the fulcrum is felt taking its proper position on the tooth, and above the alveolus, we apply the force requisite to the removal of the tooth. After we have made the turn necessary for this, and the tooth hangs, or is not entirely freed from the socket and gum, we may, by keeping our hold on the hook, disengage it, and lift it out of the mouth.

We have seen operators carefully adjust their instrument—then let go the hook and apply both hands to the handle and turn as if the head instead of the tooth was to be removed. We have a much smaller key to be used on the bicuspides, and we like it very well, using also a small hook. The adaptation and position is the same however as for the molars, but requiring much less force for their removal. We have so felt the necessity of the application we have alluded to of this instrument, that all those improvements designed for holding the hook in place, we have regarded as uncertain and objectionable, because we lose that feeling of security and certainty we have when we have hold of the hook.

In the application of this instrument to the teeth of the superior jaw, we have the head thrown far back, and stand on a stool at the back of our patient, so that we can look into the mouth and see the tooth we wish to extract; here, however, we hold the hook in place with our forefinger, still using the right or left hand as is demanded by the tooth to be extracted.

We will now state a few cases where we should change the application of this instrument.

We should do so on the lower teeth, if the tooth stood a little outside of the circle into which these teeth are generally placed. We occasionally have one tooth on either side in the inferior maxilla taking this position. The alveolus of such teeth do not point as it were to the center of the palital arch, but points much farther out, and the contiguous teeth impinge too closely to permit them to be drawn inwards. We have then another condition; the result of decay. The decay is on the labial face, and there is no hold for the hook above the alveolus. These conditions more frequently take place in the inferior than superior maxilla. We may however have the conditions above named, in the superior maxilla, and should adapt the instrument to suit the case. In these cases of decay it may be asked, where rests the fulcrum? We answer, as near the upper margin of the alveolus as it can be held; and here comes the great objections. The fulcrum must have a point to rest upon; and hence if there is decay of this kind, the fulcrum is thrown above its proper position, and we not only lose the proper application of the force for the easy removal of the tooth, but we necessarily bruise the gum. Before we leave this venerable instrument of antiquity, we will give one or two cases illustrative of the manner we have used it to advantage, even under very unfavorable circumstances.

We are not one of those who believe that every thing is smooth in dentistry and that the performance of some operations is as easy as the description.

A year or two since Dr. H. of P——— a town of some eighty miles distance in the interior of this State—after suffering violently for two or three days with pain in the anterior superior molar of the left side, had a dentist of the place attempt its extraction. After two or three efforts he broke the tooth just above the gum. He came to our city determined to have it removed. Before I saw him, he had one of our best dentists attempt its removal with the forceps. The tooth had broken above the decay—but left as was supposed, a sufficient hold above the alveolus. This hold in the last effort was broken away on the labial neck of the tooth. The Dr. having great confidence in the key called and requested us to try one on his tooth. We first cut away a portion of the alveolus on the labial side which covered the union of the roots, and with a large and pointed hook, which we forced between the roots, letting the fulcrum rest on the upper border of the alveolus and against the palital neck of the tooth which projected a very little above—we succeeded in turning it out without any difficulty. The success was here so easy that he would scarce let us apply a forcep for the removal of a lower dens sapientiæ. This however we did and somewhat redeemed the character of the instrument in his estimation. It may be said, after having removed a portion of the alveolus, could you not have removed the tooth with a forcep? We think we could. But from the repeated failures with the forceps and as we felt assured in the hands of good operators, we knew the tooth must be firmly attached and would require perhaps all the strength we had for its removal, and we felt that with the key we were certain of success. Here it will be borne in mind the hook was higher up than usual and had its proper position with reference to the fulcrum allowing this to be a little higher than usual also.

One other case and we will dismiss this instrument. A few years since a large, firmly knit, Norwegian called at our office, to have his teeth put in order. Both anterior superior molars were decayed too far to fill and for the preservation of his other teeth we advised their extraction. With a remark, do as "I thought best," he placed himself for their removal. We saw his teeth looked large and formidable and prepared ourselves accordingly. But with all the power we could exert with the forcep the tooth moved not. A second effort was alike unsuccessful. The tooth was too strong to break by proper force. We applyed our key with our usual hook. The first effort broke our hook. Here was a dilemma; but we recollected we had an old hook or two made by a smith, very large and strong, for a key of the old stamp. We selected one of these—prepared the point and fitted it to our key and with our instrument thus armed we succeeded admirably. We supposed our patient was now willing to quit, but he was of "sterner stuff" and we again tried our forcep feeling determined to test its power and he said we did not hurt him much. But it would not do—so we again fell back on the key and the old Lion never fails. We have but few such Norwegians to extract teeth for. We believe this is the only one. We had however, one Englishman who laughed at us while we were pulling at his tooth with a forcep. We threw it down and turned the laugh with our key. The first turn of our instrument the tooth flew into the middle of our office. It cracked like a pistol and we both thought it broken until we found the tooth and saw each root perfect.

We have no doubt there are those who can take out such teeth with the forcep better than we can. To such we would say they have no need of the key. We are glad to say that we meet with but few such cases. We, however, intend giving the difficulties we meet with in this operation (the extraction of teeth) and the manner of surmounting them. If any one else has a better method we hope they will give it. We regard the forcep as *the* instrument for this operation and in our next shall speak of their construction, application &c.,

J. TAYLOR.

